# Evaluation of Antimicrobial Activity of *Kitaibelia vitifolia* Extract against Proven Antibiotic-Susceptible and Multidrug-Resistant (MDR) Strains of Bacteria of Clinical Origin

**DOI:** 10.3390/plants12183236

**Published:** 2023-09-12

**Authors:** Vladimir S. Kurćubić, Svetlana V. Raketić, Jelena M. Mašković, Pavle Z. Mašković, Luka V. Kurćubić, Volker Heinz, Igor B. Tomasevic

**Affiliations:** 1 Department of Food Technology, Faculty of Agronomy, University of Kragujevac, Cara Dušana 34, 32000 Čačak, Serbia; vkurcubic@kg.ac.rs; 2 Microbiology Laboratory for Food and Water, Public Health Institute Čačak, Veselina Milikića 7, 32000 Čačak, Serbia; raketicsvetlana@gmail.com; 3 Department of Chemistry and Chemical Engineering, Faculty of Agronomy, University of Kragujevac, Cara Dušana 34, 32000 Čačak, Serbia; jelenav@kg.ac.rs (J.M.M.); pavlem@kg.ac.rs (P.Z.M.); 4 Department of Medical Microbiology, University Clinical Center of Serbia, Pasterova 2, 11000 Beograd, Serbia; kurcubiclk@gmail.com; 5 DIL German Institute of Food Technology, Prof.-von-Klitzing-Str. 7, D-49610 Quakenbrück, Germany; v.heinz@dil-ev.de; 6 Department of Animal Source Food Technology, Faculty of Agriculture, University of Belgrade, Nemanjina 6, 11080 Belgrade, Serbia

**Keywords:** *Kitaibelia vitifolia*, multidrug resistant bacteria, antibacterial activity, disc diffusion method, minimum inhibitory concentration

## Abstract

The goal of the present research was to screen the antimicrobial activity of an ethanolic extract of *Kitaibelia vitifolia* against 30 multidrug-resistant (MDR) bacterial strains isolated from healthcare-associated infections. Minimum inhibitory concentrations (MICs) of the samples against the tested bacteria were determined using the microdilution method. MDR bacterial strains were characterized using standard biochemical tests and the commercial identification systems API 20 NE and API 20 E as: *Klebsiella* spp. (18 isolates—I); methicillin-resistant *Staphylococcus aureus* (MRSA)—3; *Acinetobacter* spp.—3; *Pseudomonas aeruginosa*—5; vancomycin-resistant *Enterococcus* (VRE)—1. The sensitivity of isolated bacterial strains was determined using the disc diffusion method against 25 commonly used antibiotics. The highest level of sensitivity to *K. vitifolia* extract was confirmed in 88.89% of *Klebsiella* spp. isolates, *E. coli* ATCC 25922, two strains of MRSA (1726, 1063), *Acinetobacter* spp. strain 1578, and VRE strain 30, like *Enterococcus faecalis* ATCC 29212 (MIC =< 2.44 μg/mL). The lowest sensitivity was exhibited by 75.00% of *Acinetobacter* spp. (strains 1577 and 6401), where the highest values for MICs were noted (1250 μg/mL). The results indicate that the extract of *K. vitifolia* could be a possible source for creating new, efficient, and effective natural medicines for combat against MDR strains of bacteria.

## 1. Introduction

In routine use, antibiotics are the antimicrobial agents (AMAs) most frequently used to treat bacterial infections. Excessive, uncontrolled use of antibiotics is a prerequisite for the rapid emergence and spread of a large number of different groups of multidrug-resistant (MDR) microorganisms [1]. The phenomenon of resistance to multiple AMAs in pathogenic bacteria represents a significant threat to public health because there are very few or no available AMAs effective against infection caused by MDR strains of bacteria. Several multidrug-resistant organisms (MDROs) are commonly found in cases of healthcare-associated infections. Due to their great importance, several Gram-positive (G+) and Gram-negative (G−) bacteria have been named in the ESKAPE group (*Enterococcus faecium*, *Staphylococcus aureus*, *Klebsiella pneumoniae*, *Acinetobacter baumannii*, *Pseudomonas aeruginosa*) [2,3]. In relation to the degree of insensitivity (resistance) to antimicrobial agents, all bacteria can be divided into multidrug-resistant (MDR), extensively drug-resistant (XDR), and pan drug-resistant (PDR). Researchers believe that MDR can be defined as non-susceptibility to at least one agent from three or more AMA categories [4,5]. If the isolates of the tested bacteria are sensitive to only one or two categories of AMA, they are classified as XDR bacteria. Bacteria resistant to all known AMA, from all categories, are declared as PDR. Mutations and lateral transfer of genes have led to the increasingly common occurrence of resistance in different strains of bacteria. A group of genes has been described that directs the profile of internal resistance (“bacterial resistome”), the mutations of which make bacteria resistant to certain antibiotics without the transfer of genetic material [6]. There is evidence for rapid global dissemination of resistance to the newer generations and families of antibiotics, which have very briefly been successful against pathogenic microorganisms [7,8]. As a consequence, the present situation appears to be increasing medical costs and the mortality of patients. Inadequate prescription of antibiotics and their massive use have led to the emergence of bacterial resistance [9,10], which limits the effectiveness of even the latest generations of available drugs. Bacterial resistance represents a serious challenge because the suppression or destruction of such infectious disease agents requires the use of antibiotics that cannot be procured through routine procedures and the effects of which are questionable due to their toxicity. Thus, bacterial resistance has a very strong negative influence on the health of the wider population and the economy. For example, in the United States, over 2 million human infections with MDR microorganisms are registered annually, of which 23,000 have fatal outcomes [11].

Many of the mentioned bacteria develop resistance to multiple drugs and the infections they cause are accompanied by high rates of morbidity and mortality. It is urgent to create new antibacterial drugs capable of overcoming the various mechanisms by which bacterial strains become resistant [12,13,14,15,16,17].

Due to the dangers associated with this trend, a number of researchers have focused their research on the use of plant products in order to develop new and more effective means of combatting MDR microbial strains [18,19,20,21,22]. A wide range of secondary metabolites are abundantly available in plants for which there is evidence in in vitro assays that they have antimicrobial (AM) properties. Secondary metabolites of plants play an extremely important role in pharmaceuticals, which is indicated by the fact that about 30% of AMAs comes from products of natural origin [23,24,25,26,27]. Extracts of numerous plant species used for the prevention and treatment of illnesses caused by infectious agents have a great advantage over synthetic antibiotics due to their availability and almost no contraindications and genotoxicity [28]. The more frequent occurrence of MDR microorganisms isolated from humans and animals has revealed the urgent need to identify new and inexpensive AMAs. Many studies indicate that the products of secondary metabolism of numerous plant species possess AM activity, making plant extracts (PEs) very useful for inhibiting the occurrence of or treating infections caused by microorganisms [29,30,31]. PEs that show in vitro AM activity have limited applications because animal research and future human trails are necessary [32]. Phenolic substances, which are powerful antioxidants, are predominantly found in plant material [33]. Flavonoids, as secondary metabolites, possess significant AM potency [34]. The AM effects of different plant species are known and considered to be the result of tannins, saponins, phenolic acids, and flavonoids [35]. Even crude extracts of plant species possess strong activity against MDR strains, whereas newer generation antibiotics have limited effects. One such study finally confirmed the effect of a leaf extract of the plant species *P. betle* on confirmed G+ and G− MDR bacteria, thanks to bioactive substances that inhibited the mentioned strains [36].

Research by other authors shows that certain plant species from certain families exhibit inhibitory effects against the bacteria *S. aureus* and MRSA, and they are thought to serve as potentially effective natural AMAs [37]. According to statistics from the World Health Organization (WHO), the prevalence of MRSA is increasing worldwide and represents one of the most significant risks to public health as one of the main causes of hospitalization, which determines higher economic costs of health care [38,39]. It is imperative for healthcare institutions to implement effective infection control measures (hand hygiene, environmental cleaning) and MRSA surveillance [40,41].

Plants have an almost unlimited ability to synthesize chemically diverse, low-molecular-weight compounds through various biosynthetic pathways of secondary metabolism. The synthesis of these substances is often a defense mechanism and the plant’s response to attack by plant pathogens and the occurrence of infection (phytoalexins). If they play this role in plants, it is quite clear that these compounds will manifest protective effects under in vitro conditions as well. However, as mentioned, extrapolation to in vivo efficacy is not so simple, which is why there are few herbal medicines with confirmed effectiveness and justified application in infectious diseases. Also, despite intensive research on higher plants, the pharmaceutical industry has not discovered a potent, non-toxic compound with a broad antimicrobial spectrum that would be worth further transforming to obtain a new antibiotic [24]. Research in recent decades has defined the most important components of plants that possess AM activity. These components are also responsible for the certain effectiveness of herbal drugs. It has been confirmed that AM activity is possessed by numerous polyphenolic plant components, such as simple polyphenols, phenolic acids, lignans, quinones, flavonoids, tannins, coumarins, terpenoids, essential oils, alkaloids, lectins, polypeptides, polyacetylenes, fatty acids, and even some simple sugars or organic acids [42]. The most common secondary metabolites are flavonoids, as well as condensed tannins and gallotannins. Gallotannins are highly represented in PEs for which AM activity was reported in clinical samples [43]. Certain studies show that PEs, which are rich in polyphenolic compounds, can inhibit the reproduction of pathogens and be used as an alternative to synthetic antibiotics [44,45,46]. Flavonoids exhibit potent AM activity. It was discovered that certain alkaloids (berberine) have an inhibitory effect against selected bacterial strains. Also, certain terpenes and essential oils have been proven to possess strong AM activity [47]. Examining an ethanolic extract obtained from the aerial part of the plant species *K. vitifolia* using liquid chromatography combined with a DAD detector proved the presence of the most dominant component (rosmarinic acid) as well as slightly lower contents of p-hydroxybenzoic acid and caffeic acid. The AM activity of the ethanolic extract of *K. vitifolia* was determined using the dilution method for eight selected ATCC microbial strains and minimum inhibitory concentrations (MICs) ranging from 15.62 to 62.50 µg/mL were detected [33].

The above results were essential for continuing trials and setting the goal of the present study to investigate the antibacterial activity of an ethanolic herbal extract of *Kitaibelia vitifolia* against MDR bacterial strains isolated from healthcare-associated infections.

## 2. Results and Discussion

### 2.1. Drug Resistance Pattern of the Tested Bacterial Strains

The MDR bacterial strains were tested for antibiotic sensitivity patterns against 25 commonly used antibiotics, from different AM categories, in accordance with the recommendations of CLSI.

Results of testing the sensitivity of isolated *Klebsiella* spp. (Table 1).

Susceptibility testing of the 18 isolates of *Klebsiella* spp. to antibiotics from the carbapenems group gave the following results: 94.45% of isolates were sensitive to imipenem, 88.90% were sensitive to meropenem, and 61.11% were sensitive to ertapenem. Sensitivity to glycylcyclines—50.0% of isolates were sensitive to tigecycline. In the β-lactam + β-lactamase inhibitors group, amoxicillin-clavulonate was proven to be more efficient than piperacillin-tazobactam, and isolate sensitivity was 50.0% compared to 44.44%. In the phenicols group, sensitivity to chloramphenicol was tested, and the result was sensitivity in 44.44% of the isolates. For antibiotics from the aminoglycosides group, amikacin was the most effective, with 22.22% susceptible isolates, then netilmicin, with 16.66% susceptible isolates, and gentamicin, with only 5.55% susceptible isolates. Antibiotics from the tetracyclines group (tetracycline, doxycycline, minocycline) exhibited the identical effect, with 22.22% susceptible isolates. From the quinolones group, sensitivity to ciprofloxacin was shown by 11.10% of the surveyed strains. The isolated strains of *Klebsiella* spp. showed a slight susceptibility to cephalosporins, namely 5.55% of strains were susceptible to ceftazidime, 5.5% to cefepime, and strains showed complete insensitivity to ceftriaxone and cefuroxime. Sensitivity to trimethoprim/sulfametoxasole amounted to 5.55%. All isolates showed insensitivity to ampicillin.

*Escherichia coli* (ATCC 25922), as a control microorganism, was sensitive to all antibiotics applied across the entire categories of antimicrobials. All three tested strains of methicillin-resistant *Staphylococcus aureus* (MRSA) were resistant (100%) to the applied antibiotics in the following antimicrobial categories: aminoglycosides (gentamicin), anti-staphylococcus β-lactams or cephamycins (cefoksitin), fluoroquinolones (ciprofloxacin), generation III and IV cephalosporins (ceftazidime, cefepime, cefotaxime, ceftriaxone), and macrolides (erythromycin) (Table 2).

AM resistance is the biggest challenge to the treatment of contagious diseases. Resistance to natural and semi-synthetic antibiotics has been demonstrated as well as to synthetic substances (e.g., fluoroquinolones) or antibiotics that do not penetrate the intact cell membrane (e.g., vancomycin) [48].

From the ansamycins AM category, sensitivity to the antibiotic rifampicin was demonstrated by two MRSA strains (66.67%) and one strain was resistant (33.33%). From the folate pathway inhibitors AM category, sensitivity to the antibiotic trimethoprim/sulfamethoxazole was demonstrated by two MRSA strains (66.67%) and one strain was resistant (33.33%). From the tetracyclines AM category, tetracycline sensitivity was demonstrated by one of the examined MRSA strains (33.33%) and two strains were resistant (66.67%). One MRSA strain of those tested (33.33%) was sensitive to doxycycline and 2 strains were resistant (66.67%). Minocycline sensitivity was presented by two tested MRSA strains (66.67%) and 1 strain was resistant (33.33%). Sensitivity was exhibited by all three tested MRSA strains (100%) to the applied antibiotics in the following AM categories: oxazolidins (linezolid), phosphomycin (phosphonic acid), fusidanes (fusidic acid), glycopeptides (vankomycin), and glycylcyclines (tigecycline). Of the phenicols AM category, two strains (66.67%) were resistant to chloramphenicol and sensitivity was expressed by one of the tested strains of MRSA (33.33%). *Staphylococcus aureus* ATCC 25923, as a control microorganism, was sensitive to all antibiotics applied across the entire categories of antimicrobials.

Examining the sensitivity of three different strains of *Acinetobacter* spp. (Table 3) to the aminoglycoside antibiotics group (gentamicin, amikacin), two tested strains showed complete insensitivity. Susceptibility to tobramycin was shown by one isolate (33.33%). All three tested strains were resistant (100%) to the applied antibiotics in the following AM categories: anti-pseudomonal penicillins (piperacillin-tazobactam), anti-pseudomonal carbapanems (imipenem, meropenem), generation III and IV cephalosporins (ceftazidime, cefepime, cefotaxime, ceftriaxone), anti-pseudomonal fluoroquinolones (ciprofloxacin, levofloxacin), and penicillins + β-lactamase inhibitors (ampicillin-sulbactam). Of the folate pathway inhibitors AM category, one strain showed sensitivity to trimethoprim/sulfamethoxazole. From the tetracyclines antibiotic category, one strain showed sensitivity to tetracycline. One strain tested showed resistance and two strains were sensitive to the activities of doxycycline and minocycline (66.67%). All five of the tested clinically isolated strains of *Pseudomonas aeruginosa* (100%) were resistant to antibiotics from the following different AM categories: aminoglycosides (gentamicin, amikacin, netilmicin, tobramycin), anti-pseudomonal penicillins (piperacillin), anti-pseudomonal carbapanems (imipenem, meropenem), anti-pseudomonal cephalosporins (ceftazidime, cefepime), and anti-pseudomonal fluoroquinolones (ciprofloxacin, levofloxacin). From the polymixins AM category, four strains were sensitive to colistin (80.00%) and one isolate was resistant (20%).

The control microorganism (*Pseudomonas aeruginosa* ATCC 27853) was sensitive to all antibiotics applied across the entire categories of AMs (Table 4).

If we look at the sensitivity of the examined vankomycin-resistant Enterococcus (VRE) strain (Table 5), we note that there was absolute resistance (100%) to antibiotics from the following categories of AMs: aminoglycosides except streptomycin (gentamicin, high concentration 120 mg), streptomycin (high concentration 300 mg), fluoroquinolones (ciprofloxacin, levofloxacin), carbapanems (imipenem, meropenem), glycopeptides (vankomycin), glycylcyclines (tigecikline), tetracyclines (doxycycline, minocycline), and penicillins (ampicillin). VRE was sensitive to the oxazolidins AM category (linezolid). A control microorganism (*Enterococcus faecalis* ATCC 29212) was sensitive to all antibiotics applied.

### 2.2. Characterization of the Kitaibellia Vitifolia Plant Extract

**Figure 1:**
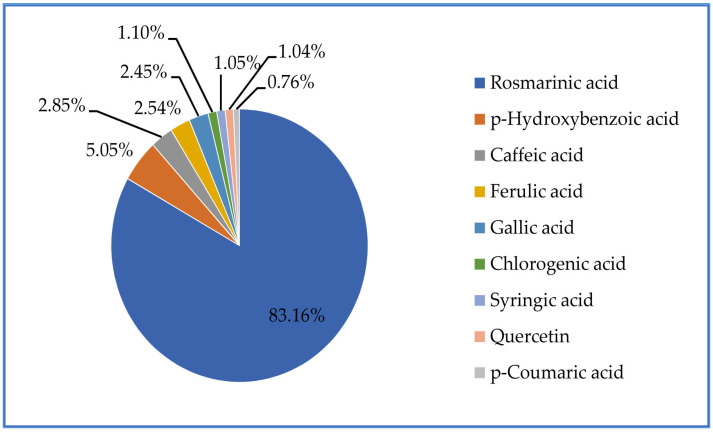
Weight percent (wt.%) of bioactive (polyphenolic) substances in the extract of the plant species *Kitaibelia vitifolia*.

**Figure 2:**
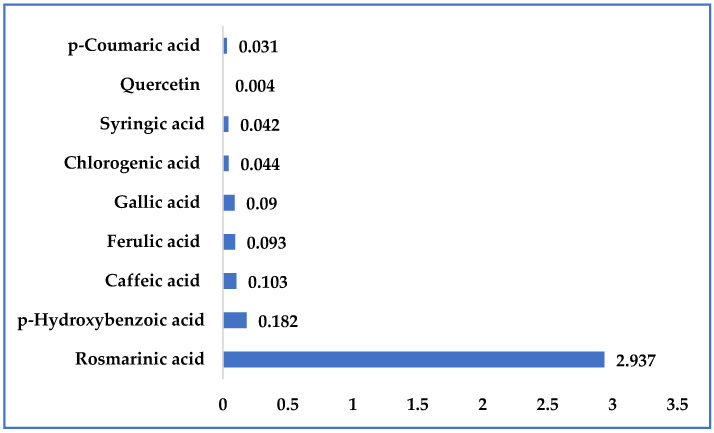
Quantitative composition of the extract of *Kitaibelia vitifolia*, expressed as concentration (mg/g of extract) of bioactive (polyphenolic) substances.

The most dominant component of the *K. vitifolia* plant extract was a phenolic acid, rosmarinic acid, which made up 83.2 wt.% of the extract (concentration 2.937 mg/g) (Figure 1 and Figure 2). Rosmarinic acid is present as a product of secondary metabolism in the extracts of different plant families (*Boraginaceae* and *Lamiaceae*). It is a natural polyphenolic antioxidant. The pure substance is a red-orange powder that is difficult to dissolve in water and dissolves easily in organic solvents. In the case of topical preparations, research was conducted to assess whether rosmarinic acid could be used as an adjuvant therapy or an alternative to steroid hormones, where it was shown that rosmarinic acid can be introduced as a new medication in the prevention of atopic dermatitis, proving that it is safe. However, the precise molecular mechanisms by which rosmarinic acid improves the skin condition of patients with atopic dermatitis are unknown [49]. In addition to rosmarinic acid, the *K. vitifolia* PE also contained other phenolic acids and the flavonoid quercetin. Among the phenolic acids, in addition to rosmarinic acid, p-hydroxybenzoic acid (5.1 wt.% of extract), caffeic acid (2.9 wt.% of extract), ferulic acid (2.6 wt.% of extract), gallic acid (2.5 wt.% *of the extract), chlorogenic acid (1.2 wt.% of the extract), singeric acid (1.1 wt.% of the extract), as well as p-coumaric acid (0.8 wt.% extract) were present in the extract. Also, the flavonoid quercetin, which was present in the examined extract at about 1.1 wt.% of the extract (Figure 1), is reported to inhibit DNA gyrase and energy metabolism [34].

Gallic acid and its methyl esters show a clear inhibitory effect against several very dangerous intestinal bacteria [50], and seven other simple phenolic acids have been found to be active against various bacteria and molds [51]. Ferulic and caffeic acids completely inhibit the growth of α-toxins produced by *Aspergillus flavus* and *Aspergillus parasiticus*.

It is assumed that phenolic acids act on the transmembrane pH gradient and membrane integrity, causing leakage of intracellular contents, interfering with transport and energy production processes, and affecting the respiratory chain [52]. Many studies have confirmed the biochemical and pharmacological activities of rosmarinic acid (antioxidant, AM, antiviral, anticancer, and antiallergic effects) [53,54]. A recent study aimed to investigate the possible role of rosmarinic acid as a potential AMA. Inhibitory effects against *C. albicans* (MIC = 0.1–0.2 mg/mL) and certain bacteria (MIC = 0.002->0.8 mg/mL) were demonstrated in the aforementioned study, which qualified rosmarinic acid as a potential innovative AMA thanks to its hemism [55]. One of the studies involved advanced testing of certain antibiotics against MDR bacteria in order to reduce the therapeutic dose and explored the AM effects and synergy of rosmarinic acid with antibiotics against *S. aureus* and MRSA, reporting a synergistic effect with commercially available antibiotics (amoxicillin, ofloxacin, and vancomycin) against S. aureus and only with vancomycin against MRSA [56].

Greater sensitivity of G− bacteria to the tested PE can be linked to the single-layered murein network in the cell wall, in contrast to G+ bacteria where the murein network is multi-layered and presents a good barrier to AM substances. The mode of action of AM agents from the tested PE depended on the type of microorganism and was mainly dependent on their cell wall structure, cytoplasmic membrane, concentration of the active substance, etc. Also, differences in AM activity have been noted, not only among different plant species, but also among the same species collected in different geographical areas and at different times, which was confirmed by previous research [57].

Polyphenolic compounds, due to their partial hydrophobicity, bind to the surface of the cell membrane of microorganisms, thus causing a change in its permeability, which enables the penetration of smaller molecules of polyphenolic compounds into the cell and further disruption of cell metabolism. Certain studies have observed the dissipation of K+ ions and ATP after treating bacteria with phenolic acids [58]. An important principle of the antibacterial (AB) activity of substances is the destruction of the energy status of the cell. We assume that at least part of these inhibitory properties is attributable to rosmarinic acid as the most dominant component in the tested PE. The AB power partly depends on the other phenolic acids present in PE.

The basic control strategies for the prevention of MDR bacteria are the vigorous development of “green” and new, efficient, and effective AB medications and the safe reformulation of existing AB drugs. The most significant obstacle to more intensive and successful development of medicinal preparations is insufficient knowledge of the principles of action of AB substances [59]. Imperative to improving results is the creation of effective routes and methods of administration of phytonutrients or their cocktails, thereby releasing AM compounds at the target site during generalized infections. The enigma is the selection of compounds that possess AM potency in phytocomplexes as well as their pharmacological reactivity. In order to achieve these necessary innovative goals, applying modern technology is inevitable, along with AM tests employing internationally standardized recognized protocols and the use of PE for which there is evidence of appropriate quality control [60,61].

### 2.3. Antibacterial Activity of the Plant Extract

The lowest resistance to the tested extract of plant species *K. vitifolia* (Table 6) was shown by isolates of *Klebsiella* spp. (88.89%, i.e., 16 out of 18 strains of *Klebsiella* spp.—MIC =< 2.44 μg/mL). The two remaining strains (11.11%) of *Klebsiella* spp. (361 and 073) showed significantly lower susceptibility (625 μg/mL). The mentioned level of the highest sensitivity to the herbal extract of *K. vitifolia* (MIC =< 2.44 μg/mL) was shown by *E.coli* ATCC 25922, two of the three strains of MRSA (1726 and 1063), *Acinetobacter* spp. strain 1578, and vankomycin-resistant *Enterococcus* (VRE) strain 30, like *Enterococcus faecalis* ATCC 29212.

*Staphylococcus aureus* ATCC 25923 and two strains of *Pseudomonas aeruginosa* (5067, 5414) exibited high susceptibility to the *K. vitifolia* plant extract (MIC = 2.44 μg/mL).

Three other analyzed isolates of *Pseudomonas aeruginosa* (1913, 1874, and 6315) revealed moderate susceptibility to the *K. vitifolia* plant extract (MIC = 156.25 μg/mL), and *Pseudomonas aeruginosa* ATCC 27853 showed the same susceptibility.

The most sensitive bacteria to the extract of plant species *K. vitifolia* (Table 6) were isolates of *Acinetobacter* spp. (75.00%, i.e., 2 of 3 strains (1577 and 6401), MIC = 1250 μg/mL). Comparative analysis revealed that the results obtained in this experiment were in agreement with the data published by other authors. Of five tested bacteria, *S. aureus* was the most sensitive to the effects of 46 extracts in their research, while *E. coli* was the most resistant [62]. It is assumed that one of the reasons for such results may be the property of G− bacteria possessing an outer membrane and periplasmic space that G+ bacteria do not [63]. It was discovered that the polyphenolic substances in sorrel extract (*H. sabdariffa*, fam. *Malvaceae*) exerted a very strong AB effect against *E. coli*. The greater the number of hydroxyl groups attached to the aromatic core, the higher the degree of hydroxylation, which strengthened AM activity [64]. Our research results were comparable to those of the studies below that were conducted using plant extracts on MDR strains of bacteria.

By comparing the results of our research with the results of phytochemical testing of *Thespesia populnea* (L.) Sol. Ex Correa [65], which belongs to the same family (*Malvaceae*), the extract of the plant species *K. vitifolia* showed significantly stronger activity. Laboratory tests of the AB activity of 7 Cameroonian extracts of edible plants against MDR G− bacteria showed MIC values from 64 to 1024 μg/mL against most of the 27 tested bacterial strains [66], on the basis of which we again concluded that the *K. vitifolia* extract in our study had a stronger AB effect.

The mode of action of each phenolic component is therefore very complex [67]. In order to improve the performance of *K. vitifolia* PE, it would be invaluable to continue researching AM potency depending on the chemical structure of each bioactive compound.

## 3. Materials and Methods

### 3.1. Plant Material

The plant species *K. vitifolia* was deposited and recognized by prof. Dr. Dmitar Lakušić, receiving identification number 16350 BEOU at the Institute of Botany of the Faculty of Biology, University of Belgrade.

### 3.2. Preparations of Plant Extract

The ethanolic extract of the aerial part of the plant species *K. vitifolia* was prepared according to the procedure carried out by Mašković et al. [33].

### 3.3. Isolation, Identification, and Characterization of Bacterial Isolates Collected from Healthcare-Associated Infections

The investigated samples were obtained from the Microbiological Laboratory of the Department of Public Health in Čačak, Republic of Serbia. A total of 30 MDR clinical bacterial strains (clinical samples of patients from the territory of Moravicki district) were isolated, identified, and characterized using standard biochemical tests and the commercial identification systems API 20 NE and API 20 E (bioMérieux, Inc, 100 Rodolphe Street, Durham, NC 27712, USA) as:

1. *Klebsiella* spp.—18 isolates (1 from blood, 17 from urine);
2. Methicillin-resistant *Staphylococcus aureus* (MRSA)—3 isolates (1 from nose, 1 from central venous catheter smear, 1 from wound);
3. *Acinetobacter* spp.—3 isolates (2 from bronchial aspirate, 1 from urine);
4. *Pseudomonas aeruginosa*—5 isolates (3 from urine, 1 from ear, 1 from bronchial aspirate);
5. Vancomycin-resistant *Enterococcus* (VRE)—1 isolate (from urine).

Standard strains for susceptibility testing of *Escherichia coli* ATCC 25922, *Staphylococcus aureus* ATCC 25923, *Pseudomonas aeruginosa* ATCC 27853, and *Enterococcus faecalis* ATCC 29212 were obtained from the American Type Culture Collection (ATCC, Manassas, VA, USA).

### 3.4. Antimicrobial Susceptibility Testing (AST) Using the Disc Diffusion (DD) Method

AST of the isolated strains and standard bacterial strains of *Escherichia coli* ATCC 25922, *Staphylococcus aureus* ATCC 25923, *Pseudomonas aeruginosa* ATCC 27853, and *Enterococcus faecalis* ATCC 29212 was performed using the DD method. AST of the bacterial strains was performed using standard AST antibiotic-impregnated discs (Bio-Rad Laboratories, 4000 Alfred Nobel Drive, Hercules, CA 94547, USA). Bacterial culture suspensions were prepared in saline at a density of 0.5 McFarland. Sterile swab suspensions were inoculated on plates containing Mueller–Hinton agar (Oxoid Limited, Wade Road, Basingstoke, Hampshire, RG24 8 PW, UK), and then the selected disks were set, up to 5 per plate, with a diameter of 90 mm. Then, incubation was carried out at a temperature of 35 °C ± 2 °C for 18 h, except for susceptibility testing of staphylococci and enterococci, for which the incubation time was extended to 24 h for detection of resistance to cefoksitin, i.e., vancomycin. At the end of the allotted time, the inhibition zones were measured and the sensitivity of bacteria was determined. The results were categorized and interpreted according to the standard in use (S, susceptible; I, intermediate; R, resistant) [68].

### 3.5. Determination of Minimum Inhibitory Concentration (MIC) Using Micro Dilution Method

The MIC of *K. vitifolia* PE for complete inhibition of the growth of selected isolated bacterial strains was determined using the microdilution method in 96-well microtiter plates covered with Mueller–Hinton broth (MHB), according to Satyajit et al. [69].

Inoculum, i.e., bacterial suspension in sterile saline, was prepared from cultures grown on non-selective agar (TSA) for 18 h. The density of the suspension was adjusted to 0.5 McFarland, except for *Enterococcus* and *Staphylococcus*, for which the initial density of the suspension was 0.8 McFarland. When the density of the suspension was expected to be 1 × 10^8^ CFU/mL, 0.1 mL of the initial suspension was transferred to a test tube with 9.9 mL of Mueller–Hinton broth. The expected number of bacteria in the suspension was 1 × 10^6^ CFU/mL. A 100 μL aliquot of this suspension was transferred to each field of the plastic microtiter plate, which already contained 100 μL of extract at a certain concentration. In this way, the number of bacteria was reduced twice, to 5 × 10^5^ CFU/mL. At the same time, the controls were set, i.e., bacterial suspension culture in the absence of extract (visible blurring and a button on the bottom of the microtiter plates after incubation) and extract without bacterial culture (absence of turbidity and sediment at the bottom after incubation).

Inoculated microtiter plates, which contained decreasing concentrations of extract and suspensions of bacteria, were incubated at 35 °C ± 2 °C for 20 h.

The MIC was the lowest concentration of AMA expressed in mg/L (μg/mL) that completely inhibited the growth of the tested strain of bacteria, that is, when there was no visible turbidity and/or sediment at the bottom of the microtiter plate.

## 4. Conclusions

The results of our research indicate that the extract of *K. vitifolia* could be a potential basis for designing and creating a new, superior, and natural AMA to combat infections caused by MDR strains of bacteria originating from hospital or public environments. We point out a particular interest and importance for its application in veterinary medicine, where the risk of MDR microorganisms is increasing day by day due to the metaphylactic approach employed primarily in the prophylaxis of respiratory diseases, as well as long-term antibiotic therapy. The focus of future research will be on the different chemical structures of the bioactive compounds constituting the extract of *K. vitifolia*, their pharmacokinetic principles, contraindicated or genotoxic effects, and everything related to the demonstrated antimicrobial effects.

## Figures and Tables

**Table 1:** Susceptibility of *Klebsiella* spp. to certain AM agents (disc diffusion method).

Strain Designation	AM Agents																								
	Gen	Ami	Net	Tob	Pip	Ert	Imi	Mer	Can	Cux	Cer	Cef	Cep	Cek	Cip	Tri	Tig	Azt	Amp	Acl	Chl	Col	Tet	Dox	Min
69	R	S	R	X	R	R	S	S	X	R	R	R	R	X	R	S	R	X	R	S	R	X	R	R	R
736	R	R	R	X	R	R	S	S	X	R	R	R	R	X	R	R	R	X	R	R	S	X	R	R	R
215	R	R	R	X	S	S	S	S	X	R	R	R	R	X	R	R	S	X	R	S	S	X	S	S	S
30	R	R	R	X	S	R	S	S	X	R	R	R	R	X	R	R	S	X	R	S	S	X	R	R	R
369	R	R	R	X	S	S	S	S	X	R	R	R	R	X	S	R	S	X	R	S	S	X	S	S	S
319	R	R	R	X	S	S	S	S	X	R	R	S	S	X	S	R	S	X	R	S	S	X	S	S	S
374	S	S	R	X	S	S	S	S	X	R	R	R	R	X	R	R	S	X	R	S	S	X	R	R	R
535	R	S	S	X	R	S	S	S	X	R	R	R	R	X	R	R	R	X	R	R	R	X	R	R	R
539	R	R	R	X	R	R	S	R	X	R	R	R	R	X	R	R	R	X	R	S	R	X	R	R	R
233	R	R	R	X	R	R	S	S	X	R	R	R	R	X	R	R	S	X	R	R	S	X	R	R	R
220	R	R	S	X	R	S	S	S	X	R	R	R	R	X	R	R	R	X	R	R	R	X	R	R	R
047	R	R	S	X	R	S	S	S	X	R	R	R	R	X	R	R	S	X	R	R	R	X	R	R	R
304	R	S	R	X	S	S	S	S	X	R	R	R	R	X	R	R	R	X	R	R	R	X	R	R	R
033	R	R	R	X	S	S	S	S	X	R	R	R	R	X	R	R	R	X	R	S	R	X	R	R	R
376	R	R	R	X	R	S	S	S	X	R	R	R	R	X	R	R	S	X	R	R	R	X	R	R	R
729	R	R	R	X	S	S	S	S	X	R	R	R	R	X	R	R	R	X	R	S	S	X	S	S	S
361	R	R	R	X	R	R	R	R	X	R	R	R	R	X	R	R	S	X	R	R	R	X	R	R	R
073	R	R	R	X	R	R	S	S	X	R	R	R	R	X	R	R	R	X	R	R	R	X	R	R	R
*E. coli* ATCC 25922	S	S	S	X	S	S	S	S	X	S	S	S	S	X	S	S	S	X	S	S	S	X	S	S	S

*Antimicrobial category*: Aminoglycosides. *Antimicrobial agents:* Gen, Gentamicin; Ami, Amikacin; Net, Netilmicin; Tob, Tobramicin; Anti-pseudomonas penicillins + β-lactamase inhibitors. Pip, Piperacillin-tazobactam; Carbapanems. Ert, Ertapenem; Imi, Imipenem; Mer, Meropenem; Generation I and II cephalosporins. Can, Cefazolin; Cux, Cefuroxime; Generation III and IV cephalosporins. Cer, Ceftriaxone; Cef, Ceftazidime; Cep, Cefepime; Cephamycins. Cek, Cefoxitin; Fluoroquinolones. Cip, Ciprofloxacin; Folate pathway inhibitors. Tri, Trimethoprim/sulfamethoxazole; Glycylcyclines. Tig, Tigecycline; Monobactams. Azt, Aztreonam; Penicillins. Amp, Ampicillin. Penicillins + β-lactamase inhibitors. Acl, Amoxicillin-clavulanate; Phenicols. Chl, Chloramphenicol; Polymixins. Col, Colistin; Tetracyclines. Tet, Tetracycline; Dox, Doxycycline, Min, Minocycline. S: Susceptible; R: Resistant; X: Not examined.

**Table 2:** Susceptibility of methicillin-resistant *Staphylococcus aureus* (MRSA) to certain AMs (disc diffusion method).

Strain Designation	AM Agents																						
	Gen	Rif	Cel	Cen	Cip	Cef	Cep	Cet	Cer	Fua	Tri	Van	Tei	Tig	Tet	Dox	Min	Dap	Ery	Lin	Chl	Pho	Qin
1726	R	S	X	R	R	R	R	R	R	S	S	S	X	S	R	R	S	X	R	S	R	S	X
1063	R	S	X	R	R	R	R	R	R	S	S	S	X	S	S	S	S	X	R	S	S	S	X
2056	R	R	X	R	R	R	R	R	R	S	R	S	X	S	R	R	R	X	R	S	R	S	X
*Staphylococcus aureus* ATCC 25923	S	S	X	S	S	S	S	S	S	S	S	S	X	S	S	S	S	X	S	S	S	S	X

*Antimicrobial category*: Aminoglycosides. *Antimicrobial agents*: Gen, Gentamicin; Ansamycins. Rif, Rifampicin. Anti-MRSA cephalosporins. Cel, Ceftaroline; Anti-staphylococcus β-lactams or cephamycins. Cen, Cefoksitin; Fluoroquinolones. Cip, Ciprofloxacin; Generation III and IV cephalosporins. Cef, Ceftazidime; Cep, Cefepime; Cet, Cefotaxime; Cer, Ceftriaxone; Fusidanes. Fua, Fusidic acid; Folate pathway inhibitors. Tri, Trimethoprim/sulfamethoxazole; Glycopeptides. Van, Vankomycin; Tei, Teicoplanin; Glycylcyclines. Tigecycline; Tetracyclines. Tet, Tetracycline; Dox, Doxycycline, Min, Minocycline. Lypopeptides. Dap, Daptomycin; Macrolides. Ery, Erythromycin; Oxazolidins. Lin, Linezolid; Phenicols. Chl, Chloramphenicol; Phosphonic acid. Pho, Phosphomycin; Streptogramins. Qin, Quinupristin/dalfopristin; S: Susceptible; R: Resistant; X: Not examined.

**Table 3:** Susceptibility of *Acinetobacter* spp. to certain AM agents (disc diffusion method).

Strain Designation	AM Agents																					
	Gen	Ami	Net	Tob	Pip	Tic	Dor	Imi	Mer	Cef	Cep	Cet	Cer	Cip	Lev	Tri	AmpS	Col	Pol	Tet	Dox	Min
1578	R	R	R	R	R	X	X	R	R	R	R	R	R	R	R	R	R	X	X	R	R	R
1577	R	R	S	R	R	X	X	R	R	R	R	R	R	R	R	S	R	X	X	R	S	S
6401	R	R	R	R	R	X	X	R	R	R	R	R	R	R	R	R	R	X	X	S	S	S

*Antimicrobial category*: Aminoglycosides. *Antimicrobial agents*: Gen, Gentamicin; Ami, Amikacin; Net, Netilmicin; Anti-pseudomonas penicillins. Pip, Piperacillin; Tic, Ticarcillin; Anti-pseudomonas carbapanems. Dor, Doripenem; Imi, Imipenem; Mer, Meropenem; Generation III and IV cephalosporins. Cef, Ceftazidime; Cep, Cefepime; Cet, Cefotaxime; Cer, Ceftriaxone; Anti-pseudomonas fluoroquinolones. Cip, Ciprofloxacin; Lev, Levofloxacin; Folate pathway inhibitors. Tri, Trimethoprim/sulfamethoxazole; Penicillins + β-lactamase inhibitors. AmpS, ampicillin-sulbactam; Polymixins. Col, Colistin; Pol, Polymyxin B; Tetracyclines. Tet, Tetracycline; Dox, Doxycycline, Min, Minocycline. S: Susceptible; R: Resistant; X: Not examined.

**Table 4:** Susceptibility of *Pseudomonas aeruginosa* to certain AM agents (disc diffusion method).

Strain Designation	AM Agents															
	Gen	Ami	Net	Tob	Pip	Tic	Dor	Imi	Mer	Cef	Cep	Cip	Lev	Azt	Col	Pol
5067	R	R	R	R	R	X	X	R	R	R	R	R	R	X	S	X
5414	R	R	R	R	R	X	X	R	R	R	R	R	R	X	R	X
1913	R	R	R	R	R	X	X	R	R	R	R	R	R	X	S	X
1874	R	R	R	R	R	X	X	R	R	R	R	R	R	X	S	X
6315	R	R	R	R	R	X	X	R	R	R	R	R	R	X	S	X
*Pseudomonas aeruginosa* ATCC 27853	S	S	S	S	S	X	X	S	S	S	S	S	S	X	S	X

*Antimicrobial category*: Aminoglycosides. *Antimicrobial agents*: Gen, Gentamicin; Ami, Amikacin; Net, Netilmicin; Tob, Tobramycin; Anti-pseudomonas penicillins. Pip, Piperacillin; Tic, Ticarcillin; Anti-pseudomonas carbapanems. Dor, Doripenem; Imi, Imipenem; Mer, Meropenem; Ant-pseudomonas cephalosporins. Cef, Ceftazidime; Cep, Cefepime; Anti-pseudomonas fluoroquinolones. Cip, Ciprofloxacin; Lev, Levofloxacin; Monobactams. Azt, Aztreonam; Polymixins. Col, Colistin; Pol, Polymyxin B. S: Susceptible; R: Resistant; X: Not examined.

**Table 5:** Susceptibility of *vankomycin-resistant Enterococcus (VRE)* to certain AM agents (disc diffusion method).

Strain Designation	AM Agents													
	Gen	Str	Cip	Lev	Dor	Imi	Mer	Van	Tei	Tig	Dox	Min	Lin	Amp
30 VRE	R	R	R	R	X	R	R	R	X	R	R	R	S	R
*Enterococcus faecalis* ATCC 29212	S	S	S	S	X	S	S	S	X	S	S	S	S	S

*Antimicrobial category*: Aminoglycosides except Streptomycin. *Antimicrobial agents*: Gen, Gentamicin, high concentration 120 mg; Str, Stremptomycin, high concentration 300 mg; Fluoroquinolones. Cip, Ciprofloxacin; Lev, Levofloxacin; Carbapenems. Dor, Doripenem; Imi, Imipenem; Mer, Meropenem; Glycopeptides. Van, Vankomycin; Tei, Teicoplanin; Glycylcyclines. Tig, Tigecycline; Tetracyclines. Dox, Doxycycline, Min, Minocycline. Oxazolidins Lin, Linezolid; Penicillins. Ampicillin; Streptogramins. S: Susceptible; R: Resistant; X: Not examined.

**Table 6:** Minimum inhibitory concentration (MIC) of *Kitaibelia vitifolia* extract.

№	Strain Designation	MIC (µg/mL)
1	69 *Klebsiella* spp.	<2.44
2	736 *Klebsiella* spp.	<2.44
3	215 *Klebsiella* spp.	<2.44
4	30 *Klebsiella* spp.	<2.44
5	369 *Klebsiella* spp.	<2.44
6	319 *Klebsiella* spp.	<2.44
7	374 *Klebsiella* spp.	<2.44
8	535 *Klebsiella* spp.	<2.44
9	539 *Klebsiella* spp.	<2.44
10	233 *Klebsiella* spp.	<2.44
11	220 *Klebsiella* spp.	<2.44
12	047 *Klebsiella* spp.	<2.44
13	304 *Klebsiella* spp.	<2.44
14	033 *Klebsiella* spp.	<2.44
15	376 *Klebsiella* spp.	<2.44
16	729 *Klebsiella* spp.	<2.44
17	361 *Klebsiella* spp.	625
18	073 *Klebsiella* spp.	625
19	*E. coli* ATCC 25922	<2.44
20	1726 MRSA	<2.44
21	1063 MRSA	<2.44
22	2056 MRSA	4.88
23	*Staphylococcus aureus* ATCC 25923	2.44
24	5067 *Pseudomonas aeruginosa*	2.44
25	5414 *Pseudomonas aeruginosa*	2.44
26	1913 *Pseudomonas aeruginosa*	156.25
27	1874 *Pseudomonas aeruginosa*	156.25
28	6315 *Pseudomonas aeruginosa*	156.25
29	*Pseudomonas aeruginosa* ATCC 27853	156.25
30	1578 *Acinetobacter* spp.	<2.44
31	1577 *Acinetobacter* spp.	1250
32	6401 *Acinetobacter* spp.	1250
33	30 vankomycin-resistant *Enterococcus* (VRE)	<2.44
34	*Enterococcus faecalis* ATCC 29212	<2.44

## Data Availability

Data sharing is not applicable to this article.
